# Prevalence of Opioid Dependence and Opioid Agonist Treatment in the Berlin Custodial Setting: A Cross-Sectional Study

**DOI:** 10.3389/fpsyt.2020.00794

**Published:** 2020-08-12

**Authors:** Kira von Bernuth, Peter Seidel, Julia Krebs, Marc Lehmann, Britta Neumann, Norbert Konrad, Annette Opitz-Welke

**Affiliations:** ^1^Institute of Forensic Psychiatry, Charité-Universitätsmedizin Berlin, corporate member of Freie Universität Berlin and Humboldt-Universität zu Berlin, Berlin, Germany; ^2^Department of Psychiatry and Psychotherapy, Prison Hospital Berlin, Berlin, Germany; ^3^Prison Hospital Berlin, Plötzensee Prison, Berlin, Germany

**Keywords:** opioid dependence, opioid agonist treatment, prison, prison health care, substitution substances, treatment access, treatment variability

## Abstract

**Background:**

Among people living in detention, substance use is highly prevalent, including opioid dependence. Opioid agonist treatment (OAT) has been established as an evidence-based, first-line treatment for opioid dependence. Despite high prevalence of opioid dependence, conclusive data regarding its prevalence and the OAT practice in German prisons is scarce; rather, the existing data widely diverges concerning the rates of people in detention receiving OAT.

**Materials and Methods:**

We conducted a cross-sectional survey of all detention facilities in Berlin. On the date of data collection, a full census of the routine records was completed based on the medical documentation system. For each opioid dependent individual, we extracted sociodemographic data (i.e., age, sex, and non-/German nationality, whether people experienced language-related communication barriers), information about OAT, comorbidities (HIV, hepatitis C, schizophrenia), and the detention center, as well as the anticipated imprisonment duration and sentence type. The data was first analyzed descriptively and secondly in an evaluative-analytical manner by analyzing factors that influence the access to OAT of people living in detention.

**Results:**

Among the 4,038 people in detention in the Berlin custodial setting under investigation, we identified a 16% prevalence of opioid dependence. Of the opioid-dependent individuals, 42% received OAT; 31% were treated with methadone, 55% were treated with levomethadone, and 14% were treated with buprenorphine. Access to OAT seemed mainly dependent upon initial receipt of OAT at the time of imprisonment, detention duration, the prisons in which individuals were detained, German nationality, and sex. The overall prevalence of HIV was 4–8%, hepatitis C was 31–42%, and schizophrenia was 5%.

**Conclusions:**

The prevalence of opioid dependence and access to OAT remains a major health issue in the custodial setting. OAT implementation must be especially intensified among male, non-German, opioid-dependent individuals with a short detention period. Treatment itself must be diversified regarding the substances used for OAT, and institutional treatment differences suggest the need for a consistent treatment approach and the standardized implementation of treatment guidelines within local prison’s standard operating procedures. Testing for infectious diseases should be intensified among opioid-dependent people living in detention to address scarcely known infection statuses and high infection rates.

## Introduction

In 2007, the World Health Organization (WHO) identified dependence on drugs, alcohol, or tobacco as being among the most common physical illnesses in the worldwide prison healthcare practice, alongside infections, dental diseases, and chronic disorders (1). This condition also applies to the German custodial setting; according to current estimates, 20–50% of people living in German prisons are addicted to alcohol, 70–85% are nicotine dependent, and 20% are opioid dependent (2). The WHO recommends and recognizes opioid agonist treatment (OAT) as a fundamental, evidence-based method in treating opioid dependence (3). The German Association for Addiction Medicine suggests agonist treatment as a first-line treatment for diagnosed opioid dependence (4) because it reduces mortality (5–7), decreases heroin use, and increases the number of patients retained in treatment (8, 9). Furthermore, OAT affects the transmission of infectious diseases by reducing the prevalence of injection drug use (IDU) (10–12) as well as the risk of hepatitis C and HIV acquisition (13–16). Several recent studies point out that these results may also be transferred to the custodial setting (17–20).

Nevertheless, OAT remains a controversial topic in the prison healthcare sector. OAT is available in nearly all prisons of Western European countries, but it is often provided under more restrictive conditions than those present in the broader community (21). Even if Germany was one of the first European countries to implement OAT in the custodial setting, it still had remarkably low rates of prison population receiving OAT twenty years later (22), while the prevalence of IDU among people in detention is estimated higher compared to other European countries (23). Moreover, as in other European countries, high variability exists in treatment practice on a national level (21, 24). OAT practice in German prisons even subject to trial in front of the European Court of Human Rights in 2016 (25). Subsequently, the German state was condemned for not fulfilling its obligation to provide independent medical expertise to determine whether or not the provision of OAT was necessary (26).

Despite its political impact and high prevalence of opioid dependence, conclusive data regarding prevalence and the OAT practice in German prisons is scarce. Thus, the estimated prevalence of opioid dependence in prisons considerably varies depending on the source (27–29). The estimates concerning the rates of people in detention receiving OAT are similarly heterogeneous. In 2017, a large-scale secondary data analysis of pharmacy sales data estimated that, on a national level, merely 10% of all opioid-dependent people in detention received adequate substitution treatment but also mentioned the high variability between the various federal states (24). Meanwhile, the results of the national report concerning substance-related dependence problems suggest that, in Berlin, 52% of all opioid-dependent people in detention receive substitution treatment (29).

Even if the variability of OAT implementation is emphasized vividly by these numbers, only few studies focus on the question which criteria are used in the prison health care practice to admit individuals to OAT. Scientific literature emphasizes the role of an existing OAT at the time of imprisonment; it seems to be a main criterion for access to treatment during detention (27, 30, 31). Further, some authors discuss that access to OAT depends on infection with HIV and hepatitis C (27, 32). This may derive from the evolution of OAT where the treatment was amongst others first made available to individuals with infectious diseases (33). Further, a German-wide study that questioned prison physicians about prevalence of opioid dependence and availability of OAT suggested that people living in detention with diagnosed psychosis were more likely to access OAT, probably in order to achieve mental and psychiatric stability (27). Additionally, the detention duration is considered a critical variable in individuals’ access to OAT but is contradictorily discussed. Some authors argue that agonist treatment is more likely to be granted to individuals with short-term imprisonment (27, 30, 33) while other works argue that individuals with a sentence below two years are more likely to be confronted with an abstinence-oriented approach (34). More generally, language barriers seem to have an impact on individuals’ access to addiction treatments outside prison (35, 36); a fact that most probably also applies to the custodial setting.

### Aims

Our work aimed primarily to identify the prevalence of opioid dependence and OAT in the custodial setting in Berlin and to assess the actual OAT practice regarding substances used for OAT. Further, we aimed to identify factors that affect individuals’ access to OAT in prison.

## Materials and Methods

### Setting

We conducted the survey in the Berlin custodial setting, which comprises six prisons, the youth custody center, and the prison hospital of Berlin. On the date of data collection, 4,038 people were living in detention. Sentence types included penal incarceration, pre-trial detention, juvenile sentence, and compensation imprisonment, the last of which is a form of imprisonment assigned to individuals who are “unwilling or unable to pay a fine for committing a criminal offence” (37). In each prison, a physician is responsible for the entrance examination and primary healthcare services (2). OAT is executed by either general practitioners with additional qualifications in addiction medicine or by psychiatrists. All medical interventions performed during detention are to be documented in the medical section of the electronic documentation system called Basis-Web.

### Design

On March 25, 2019, we conducted a cross-sectional survey and extracted data from the routine records of the 4,038 people in detention recorded in the medical documentation system. Prior to analysis, all cases were assigned pseudonyms so that no connections could be made between cases and the individuals’ names.

### Patient and Treatment Information

We used the documentation system’s integrated, advanced search mode to extract for each detention facility separately all files marked with either the terms “BTM” (meaning Betäubungsmittel; an abbreviation for the German term for narcotics), substitution, detoxification, addiction disease, long-term substitution, drug addiction, tapered withdrawal, opioid dependence (corresponding with F11.2 in the International Classification of Diseases), or polyvalent substance use disorder (corresponding with F19.2 in the International Classification of Diseases) (38). Subsequently, we individually investigated the identified files for documented opioid dependence, as not every detention center used the same markers and not every marker exclusively referred to opioid dependence. Individuals were defined as opioid dependent if a medicinal prescription for OAT or withdrawal therapy was documented in their files. That means we focused on opioid dependence during imprisonment and not on a lifetime prevalence of opioid dependence. In our clinical routine, we experience that individuals directly mention substance use towards medical staff, which facilitates diagnosing substance dependence. We therefore relied on the detection of opioid dependent individuals through the documentation system, even if some individuals may have passed undiagnosed if they did not mention opioid dependence during diagnostic interviews. Duplications due to files marked with more than one term were eliminated. We included polydrug use in the search categories, since in the clinical routine, the diagnosis is also assigned to patients who mainly consume opioids alongside a varied co-usage of additional substances. We did not include dependence upon substances mainly used for pain management, such as fentanyl or tramadol, as this concerns only a minority of opioid users in Germany (39), probably due to restrictive prescription politics (40). We thus extracted 652 people living in detention with documented opioid dependence.

We obtained sociodemographic data for each individual with reported opioid dependence including age, sex, non-/German nationality. We extracted information about their OAT including the prescribed substance, if OAT was begun prior to imprisonment, and if OAT was begun or terminated during imprisonment. Tapered withdrawal with opioids was not considered an OAT. For information about the detention setting, we extracted the prison, the anticipated imprisonment duration, and the sentence type for each individual. Fifteen opioid-dependent individuals were in preventive detention or life imprisonment; in these cases, their estimated duration was not defined. For statistical reasons, we therefore labeled the detention length using the reports of the German Institute of Criminology (41, 42). Furthermore, we recorded each individual’s infection status for HIV and hepatitis C. “No infectious disease”/”no HIV/hepatitis C” noted in the entrance examination or documented negative test results were reported as no infection. Anamnestic infection or positive test results were reported as infection. If neither was documented, the status was reported as unknown. The comorbidity of schizophrenia was also extracted. We analyzed the schizophrenia diagnosis rather than psychosis because the latter was not documented consistently. Even if by this manner we could not verify the influence of psychosis on the access to OAT as assumed by Schulte et al. (27), we nevertheless included schizophrenia in the model as we assumed from our experience that schizophrenic individuals may experience barriers to access treatment due to their diagnosis. Finally, language-related communication barriers were analyzed. If the anamnesis contained the term “yes”, “good”, or “sufficient”, or if no annotation was made about an individual’s language skills, we recorded “no communication barriers”, which also signified that the physician and patient may have had another common language apart from German. Documentation of the term “no”, “some”, “language barrier”, “little”, or “broken” in reference to language skills was reported as a communication barrier.

Approval for the research was obtained from both the Criminological Service of the Law Enforcement Agency of Berlin (KrimD 45/3/009/19) and the local ethics committee at Charité-Universitätsmedizin (EA1/082/19).

### Statistical Analysis

For the 652 diagnosed opioid users, we computed general descriptive statistics for their sociodemographic data and prevalence. The continuous parameters of age and estimated detention length are presented respectively as the arithmetic mean plus the standard deviation and the median plus the interquartile range. Categorical parameters are indicated as absolute frequencies and percentages. We formed sub-groups and compared central tendencies of the continuous variables using the Mann-Whitney test for the variable detention length (no normality assumption) and an independent t-test for the variable age (normality assumption) (43). Categorical parameters were compared using Pearson’s Chi-square test.

In order to identify the factors that statistically correlated with the provision of OAT, we calculated binary logistic regressions. The factors age, sex, non-/German nationality, language-related communication barriers, schizophrenia, hepatitis C, HIV, receipt of OAT prior to imprisonment, detention duration, prison, and sentence type were included as independent variables to assess their impact on the receipt of OAT during detention. As the youth custody center offered no OAT, it was excluded from the regression models; the women’s prison was additionally excluded because sex was a variable. Thus, a total of 641 people in detention were included in the models. The variables of hepatitis C or HIV infection and estimated detention length achieved missing values, and therefore we applied multiple imputation (m = 20 imputations). We included the previously defined independent and dependent variables as well as the respective outcomes in the imputation model.

For all analyses, p <.05 was considered significant. We performed the analyses using IBM SPSS Statistics, version 25 and DB-Browser for SQLite, version 3.11.2.

## Results

### Prevalence of Opioid Dependence

On March 25, 2019, 4,038 people were detained in the Berlin custodial setting. Of these individuals, 652 were documented as opioid dependent, thus representing 16% of the prison population (see **Table 1**). The prevalence of documented opioid dependence varied between 3% (n = 18/631) in a day-release prison and 25% (n = 211/857) in prison A.

**Table 1:** People living in detention and opioid dependence in the custodial setting in Berlin (March 2019), *data is shown as n (%)*.

		Prison A	Prison B	Prison C	Women’s prison	Prison D	Prison for day release	Youth custody center	Total
People living in detention		857	957	594	233	488	631	278	**4,038**
Opioid dependent people in detention		211 (25%)	153 (16%)	139 (23%)	51 (22%)	69 (14%)	18 (3%)	11 (11%)	**652 (16%)**
*The following percentages refer to the number of opioid dependent individuals*									
Individuals receiving OAT		108 (51%)	32 (20%)	55 (40%)	43 (84%)	26 (38%)	10 (56%)	0	**274 (42%)**
Substances used for OAT	Methadone (%)	23 (21%)	29 (91%)	11 (20%)	12 (28%)	10 (38%)	0	–	**85 (31%)**
	Levomethadone (%)	67 (62%)	0	39 (71%)	23 (53%)	15 (58%)	7 (70%)	–	**151 (55%)**
	Buprenorphine (%)	18 (17%)	3 (9%)	5 (9%)	8 (19%)	1 (4%)	3 (30%)	–	**38 (14%)**

### Prevalence of OAT and Course Details

Of the 652 opioid-dependent people in detention, 274 received OAT (42%; n = 274/652). All detention facilities except the youth custody center provided OAT and the substitution rate varied between 20% (n = 32/153) in prison B and 84% (n = 43/51) in the women’s prison (see **Table 1**).

A total of 202 individuals were already receiving OAT at the time of their imprisonment; of those treatments, 73% (n = 147/202) were continued without any interruption until the date of data collection (see **Table 2**), while 16% (n = 33/202) were ended at some point during detention and 11% (n = 22/202) at the beginning of imprisonment. As no OAT was provided in the youth custody center, one individual who received OAT at the time of imprisonment stopped receiving treatment. At the women’s prison, all OATs prior to imprisonment were continued until the date of data collection.

**Table 2:** Baseline characteristics of opioid dependent individuals (N=652) in the custodial setting in Berlin (March 2019), data is shown as n (%), age as mean [SD, standard deviation], and length of detention period as median [IQR, interquartile range].

Sex	Male	601 (92%)
	Female	51 (8%)
Age [years]		37 [SD = 8]
Nationality	German	323 (50%)
	Non-German	329 (50%)
Language-related communication barriers	No	508 (78%)
	Yes	144 (22%)
Estimated detention duration [months]		17 [IQR = 25]
Sentence type	Penal incarceration	458 (70%)
	Compensation imprisonment	95 (15%)
	Pretrial detention	99 (15%)
Current OAT	Total	274 (42%)
	OAT at time of imprisonment	159 (58%)
	OAT begun during detention	115 (42%)
Continuity of OAT previous to detention	Continued	147 (73%)
	Ended during detention	33 (16%)
	Ended at beginning of detention	22 (11%)

The people receiving OAT during detention and the individuals without OAT differed statistically significantly regarding age, nationality, and estimated detention length (see **Table 3**).

**Table 3:** Descriptive data from the group receiving OAT and the group without treatment at the day of data collection, *data is shown as n (%), age as mean [SD] and length of detention period as median [IQR]*.

			OAT (n = 274)	no OAT (n = 378)	p value
Sex		Male	231 (84%)	370 (98%)	<.000
		Female	43 (16%)	8 (2%)	
Age [years]			39 [SD = 8]	36 [SD = 8]	<.000
Nationality		German	190 (69%)	133 (35%)	<.000
		Non-German	84 (31%)	245 (65%)
Language-related communication barriers		no	249 (91%)	259 (68%)	<.000
		yes	25 (9%)	119 (32%)
Estimated detention duration [months]			21 [IQR = 30]	15 [IQR = 23]	.001
Sentence type		Penal incarceration	204 (74%)	254 (67%)	.045
		Pretrial detention	51 (17%)	44 (12%)	.013
		Compensation imprisonment	19 (7%)	80 (21%)	<.000

**Figure 1:**
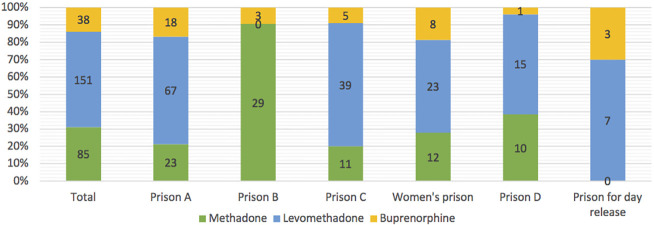
Substances used for OAT in the Berlin custodial setting, *data is shown as n*.

Three different substances were prescribed for OAT; 31% of individuals (n = 85/274) were treated with methadone, 55% (n = 151/274) with levomethadone, and 14% (n = 38/274) with buprenorphine (see **Table 1**). The number of prescribed substances varied widely among the different custodial facilities, ranging from 0–91% for methadone, 0–71% for levomethadone, and 4–30% for buprenorphine (see **Figure 1**).

### Prevalence of HIV, and Hepatitis C and Schizophrenia

The hepatitis C status was unknown for 27% (n = 176/652) and the HIV status for 43% (n = 280/652) of the opioid dependent people in detention. The infection status of both HIV and hepatitis C was known for 56% (n = 364/652) of the opioid-dependent individuals, while in 26% (n = 168/652) of the records, no information was entered for either. The documented infection status differed significantly between the subgroups (see **Table 4**).

**Table 4:** Prevalence of HIV, HCV and schizophrenia among opioid dependent individuals in the overall prison population and different subgroups, *data is shown as n (%)*.

	Total (n = 652)	Male (n = 601)	Female (n = 51)	p value	OAT (n = 274)	no OAT (n = 378)	p value
Both HIV and HCV status known	364 (56%)	344 (57%)	20 (39%)	.013	182 (66%)	182 (48%)	.000
Both HIV and HCV status unknown	168 (26%)	148 (25%)	20 (39%)	.022	47 (17%)	121 (32%)	.000
HCV infection*	199 (31/42%)	177 (29/40%)	22 (43/70%)	.042/.001	100 (36/45%)	99 (26/39%)	.005/.207
HIV infection*	28 (4/8%)	23 (4/7%)	5 (10/25%)	.043/.002	16 (6/9%)	12 (3/6%)	.098/.432
Schizophrenia	30 (5%)	29 (5%)	1 (2%)	.349	11 (4%)	19 (5%)	.543

*Prevalence is shown once related to the respective group/once related to the number of individuals for which the respective infection status was known.

We therefore calculated the prevalence of HIV and hepatitis C twice: once related to the overall number of opioid-dependent people in detention and once related to the number of people in detention for which the respective infection status was known. Thus, the overall HIV prevalence was 4% (n = 28/652) and 8% (n = 28/372; see **Table 4**), respectively, while the overall prevalence of hepatitis C was 31% (n = 199/652) and 42% (n = 199/476), respectively.

Thirty schizophrenia cases were reported, which amounted to an overall prevalence of 5% (n = 30/652) among opioid-dependent people in detention (see **Table 4**).

### Impacts on OAT Provision

In the binominal logistic regression model, the most significant predictors of OAT provision were female sex [adjusted odds ratio: 28.575, (95%, CI: 9.057–90.156), p <.000], German nationality [2.170, (1.330–3.539), p = .002], receipt of OAT at the time of imprisonment [12.071, (7.175–20.308), p <.000], estimated detention duration [1.012, (1.005–1.018), p = .001], compensation imprisonment status [3.383, (1.675–6.833), p = .001], as well as detention in prison A [6.285, (2.728–14.478), p <.000] and prison C [3.840, (1.611–9.153), p = .002; see **Table 5**]. Hepatitis C infection had a statistically significant impact in the model only preceding imputation [1.977, (1.069–.657), p = .030].

**Table 5:** Association between potential factors and receiving OAT from binary logistic regression after imputation.

		Coefficient	Standard error	p value	Adjusted odds ratio	BCa 95% Confidence Interval	
						Lower	Upper
Age		.009	.015	.555	1.009	.980	1.038
Sex (female)		3.353	.586	.000	28.575	9.057	90.156
German nationality		.775	.250	.002	2.170	1.330	3.539
No language-related communication barriers		.659	.351	.060	1.933	.971	3.847
Schizophrenia		.409	.495	.409	1.505	.570	3.971
HCV infection		.288	.263	.275	1.333	.795	2.236
HIV infection		.029	.461	.950	1.029	.415	2.550
Receipt of OAT at the time of imprisonment		2.491	.265	.000	12.071	7.175	20.308
Estimated detention duration [months]		.012	.003	.001	1.012	1.005	1.018
Sentence type	Penal incarceration	1 (reference)					
	Pretrial detention	.488	.617	.429	1.629	.486	5.459
	Compensation imprisonment	1.219	.359	.001	3.383	1.675	6.833
Prison	Prison D	1 (reference)					
	Prison for day-release	.310	.681	.649	1.363	.359	5.182
	Prison A	1.838	.426	.000	6.285	2.728	14.478
	Prison C	1.345	.443	.002	3.840	1.611	9.153
	Prison B	.396	.561	.479	1.487	.495	4.461
Constant		-4.369	.738	.000	.013	.003	.054

R² = [.485–.490] (Nagelkerke) [.067–.745] (Hosmer & Lemeshow). Model χ²(15) = [258.443–262.042], p < .000.

## Discussion

### Prevalence of Opioid Dependence and OAT

With a 16% prevalence of opioid dependence, our results reveal a lower rate than do previously conducted studies, which estimated the prevalence of current or former IDU at 21.9–29.7% among people living in German prisons (27, 28). This discrepancy may have been influenced by our definition of opioid dependence, which contrasts with other studies in that it focuses on actual opioid consumption at the time of imprisonment rather than a lifetime incidence of substance injection. Further, it could reflect the overall decrease of IDU observed in European countries (44) as the data from the previously cited studies is more than ten years old. More generally, this result fits within the estimated range of prevalence at 2–38% for IDU in European prisons (21). The relation of non-German to German opioid-dependent people in detention corresponds with those in the overall Berlin custodial setting, as about half of the people living in Berlin prisons are not of German nationality (45). As a whole, the results again point out that opioid dependence is more frequent in the custodial setting than in the community, where it is estimated at around 3.1/1000 in Berlin as well as in Germany (46).

Our observed OAT rate of 42% is significantly higher than the estimated 10% of dependent individuals in detention receiving OAT on the national level (47). The OAT rate in the community can be estimated at 48% in Berlin (46, 48) and in 2012, the European Monitoring Center for Drugs and Drug Addiction estimated that at least one in two of the estimated population of problem opioid users in Europe receive OAT (49). These statistics suggest that an OAT rate of 42% in the Berlin custodial setting, especially with a variability between 0–84%, is still rather low compared to the extramural practice. One may assert that not all people in detention eligible for OAT are willing to begin an agonist treatment. Even if this were true, the results of a study conducted in a remand prison in Switzerland suggest that opioid-dependent individuals who are entering detention are highly willing to begin OAT (50). This implication suggests that an OAT rate of 42% is not necessarily due to an individuals’ lack of interest, but rather may reflect the ineffective implementation of OAT in the custodial setting.

### Substances Used During OAT

Contrarily to the extramural setting, only three substances were administered alongside individuals’ OATs in the Berlin prisons. Compared to the extramural practice based on the statistics of the annual German Report on Drugs and Addiction (48), we observed that methadone and buprenorphine were used less often and levomethadone more often. Despite being used in the extramural setting (48), codeine, dihydrocodeine, diamorphine, and retarded morphine are not offered in Berlin prisons. Though, different treatment substances create the opportunity to more efficiently address individual physical or mental adverse effects and differences in metabolization (51, 52). As Kourounis et al. have determined, a lack of pluralism in medication options creates a treatment design barrier that makes it more difficult for patients receiving OAT to remain in treatment (53). Thus, the use of only two different substances in some prisons may reflect a restricted prescription practice in prison that may present a significant treatment design barrier.

### Access to OAT During Detention

The fact that existing OAT at the time of imprisonment had a major impact on the access to OAT during detention aligns with findings from the German and European level (27, 30, 31). However, a study conducted at the German national level found that 70% of individuals undergoing OAT at the time of imprisonment were required to end their treatment upon incarceration (54) while our results show that 73% of treatments that began in the extramural system were continued until the date of data collection. This discrepancy may reflect the differences in the OAT practices at the transition from the extramural to the intramural sector across various federal states. For instance, in Saarland, no prisons are supplied with OAT medicines, and in Lower Saxony, all prisons are supplied with such substances (24).

Our research demonstrated lower rates of opioid dependence among women than previous studies, which estimated dependence on opioids between 27–50% in the women’s custodial setting (55, 56). However, we found similar to higher rates of OAT provision which varied between 13–84% in other works (2, 55). It is to note that the rate of opioid dependence in the women’s prison was still among the highest in our study. Further a study previously conducted in the custodial setting in Berlin pointed out that among women with addiction living in prison, 90% had at least one other mental disorder (55). This shows that opioid dependent women remain a small, but vulnerable group in the prison setting, which needs to be addressed by prison health services.

Furthermore, we were surprised by the impact of the German nationality on treatment access. In the extramural system, access to OAT is essentially dependent upon individuals’ access to healthcare, which is closely associated with nationality and legal residency status. Assuming that German nationality is an indicator for individuals’ health insurance status, the extramural health insurance situation still seems to influence their intramural access to treatment. This is even more striking considering that healthcare costs during detention are covered by the federal states (57).

We observed a significantly higher share of people in detention with language barriers among those without OAT than among those who received treatment. This finding suggests that communication abilities still have a practical impact on individuals’ receipt of OAT.

In contrast with the findings of other studies (27, 32) and our expectations, we found that HIV and/or hepatitis C infection did not seem to be a predictor for the provision of OAT during detention. This result may be explained by the fact that both the HIV and hepatitis C infection statuses were exclusively known for 56% of people in detention, which thus renders rather unlikely a systematic decision regarding whether or not individuals should begin OAT depending on their infection status. Furthermore, the fact that infection status was documented significantly more often among individuals who received OAT during detention suggests that an individual’s receipt of OAT is associated with a higher rate of proposed testing for HIV and hepatitis C. Meanwhile, among people in detention worldwide, HIV and hepatitis C prevalence is estimated at 3.8 and 15.1%, respectively (58). A recent German study found that 66% of individuals who inject drugs are infected with hepatitis C and 4.9% with HIV (39). We determined similar results with an estimated prevalence of 4–8% for HIV and 31–42% for hepatitis C among the opioid dependent individuals, showing that both HIV and hepatitis C still present a major health issue in the custodial setting. Infection status seems to be less often known among male opioid dependent individuals without OAT and among women, while in our results these even presented higher infection rates than men of both HIV and hepatitis C. The controlled structure of imprisonment should be used to systematically propose testing, counselling and treatment of infectious diseases (39). The supply of OAT should be intensified as a strategy of harm reduction among others, in order to prevent new infections among people in detention (17, 19, 20, 39).

Contrary to our expectations, diagnosed schizophrenia had no statistical impact on provision of OAT. However, we identified a 5% prevalence for schizophrenia among opioid-dependent people in detention. As such, schizophrenia remains an important comorbidity, as its prevalence is higher herein than in the overall population, where it is estimated at 3.1% (59).

The fact that each month of detention increased a person’s likelihood to receive OAT may reflect the attitudes of physicians who prefer to administer OAT to individuals with longer sentences in order to assure the treatment’s stability and durability. Meanwhile, the WHO recommends to propose OAT to people in detention who are not yet receiving such treatment even if the remainder of their sentence is short, as OAT reduces both the risk of overdose after release and reincarceration rates (3) and this further could reduce infection rates with hepatitis C (17).

Eventually, we observed that the access to OAT seemed to depend on the prison in which individuals were detained. It is noteworthy that prison D unites two different custodial facilities with two different medical entities, one of which primarily detains individuals under compensation imprisonment. Thus, compensation imprisonment represented a predictor for OAT most likely due to factors related to this sub-prison. Several authors discuss differences in attitudes held toward liberal and harm-reduction drug politics—which translate into different institutional policies and regulations—as a main reason for the general hesitation to use OAT in prisons and its high implementation variability between different federal states (24, 30, 34, 60). Yet the variability of implementation seems not only to be limited to the national level (24), but also to apply to the federal state level. This variability in treatment implementation at every institutional level—within countries and federal states—has been observed in other European countries (33). It suggests that different prisons have different OAT practices and that indications for agonist treatment do not follow a common approach. Another reason for the variability of implementation may be differences in the respective prison physicians’ qualifications in addiction medicine. This observation is even more striking considering that Berlin represents a federal state and a city at the same time. It could have been assumed that the geographical closeness and the institutional frame would lead to a consistent treatment approach. It is important to consider that, even if OAT implementation in prison presents certain limitations due to institutional implications, it remains a setting that may theoretically offer a low-threshold service for drug users regarding accessibility barriers (53). The German federal state of North Rhine-Westphalia has recently shown that the amount of people in detention receiving OAT could significantly be increased by a clear statement of the Ministry of Justice that OAT has to be implemented in prisons as well as treatment recommendations developed by the medical profession defining a standard of care, medical education of prison doctors and a monitoring system (61). Systematically offering OAT through primary healthcare, based on existing international and national treatment guidelines for opioid dependence (3, 4), would reduce selective intake criteria and consequently improve accessibility. Other European countries have demonstrated that this approach is both possible and generally well-accepted by people living in detention (50, 62).

### Limitations

When interpreting our findings, it must be noted that all results are as valid as the documentation provided for analysis. While the people in detention receiving OAT were quite thoroughly documented, individuals who did not mention opioid dependence to medical staff during the entrance examination or during detention did not appear in our analysis. It is likely that we overestimated the rate of individuals receiving agonist treatment.

Further, it must be considered that the documentation system used in the Berlin prison setting was not designed for epidemiological analysis; this fact most importantly relates to the estimated prevalence of HIV and hepatitis C. As mentioned above, the infection statuses were not consistently documented in the electronic system and we had to rely on documented test results as well as on anamnestic information. Though, documented positive or negative test results are more useful than anamnestic information of “no infectious disease”, as this statement may imply a summary of negative results but could also be a simple re-statement of unconfirmed medical history. Such, each calculated prevalence and its impact on individuals’ access to OAT merely present an approximation.

Eventually, we conducted quantitative, cross-sectional research that cannot explain all of our findings and does not display long-term outcomes. Individuals’ perspectives of substitution treatment remain unknown and are necessary to consider if we are accurately to assess their needs and experienced barriers to accessing OAT in prison. Similarly, institutional factors of the prison setting that affect OAT implementation are only marginally represented in our study. Our results can therefore be considered a first quantifying step that necessitates further qualitative research.

## Conclusion

Our results reveal that opioid dependence remains a major health issue in the custodial setting that must be further addressed. By comparing prisons in Berlin to the German extramural setting, OAT seems to be implemented less often in the former. OAT implementation in prisons must be intensified and treatment itself diversified regarding the substances used during OAT, especially among male, non-German, opioid-dependent individuals with a short detention period. The prison in which individuals are detained has a major impact on OAT implementation, which suggests that institutional changes are needed in order to implement a consistent treatment approach on a federal state level—such as treatment guidelines for opioid dependence- within local prison’s standard operating procedures. Such an approach is even more severely needed considering that OAT is a measure facing high infection rates of HIV and hepatitis C among opioid-dependent people living in prison.

## Data Availability Statement

The datasets generated for this study are available on request to the corresponding author.

## Ethics Statement

The studies involving human participants were reviewed and approved by Local ethics committee at Charité-Universitätsmedizin. Written informed consent for participation was not required for this study in accordance with the national legislation and the institutional requirements.

## Author Contributions

KB, AO-W, JK, and NK designed the study. KB, PS, ML, and BN collected the data. KB, JK, NK, and AO-W analyzed and interpreted the data. KB, BN, and AO-W wrote the final draft of the manuscript. KB, NK, and AO-W had full access to all the data in the study and take responsibility for the integrity of the data and the accuracy of data analysis. All authors contributed to the article and approved the submitted version.

## Conflict of Interest

The authors declare that the research was conducted in the absence of any commercial or financial relationships that could be construed as a potential conflict of interest.
